# Post-stroke Neurogenesis: Friend or Foe?

**DOI:** 10.3389/fcell.2021.657846

**Published:** 2021-03-23

**Authors:** María Isabel Cuartero, Alicia García-Culebras, Cristina Torres-López, Violeta Medina, Enrique Fraga, Sandra Vázquez-Reyes, Tania Jareño-Flores, Juan M. García-Segura, Ignacio Lizasoain, María Ángeles Moro

**Affiliations:** ^1^Neurovascular Pathophysiology, Centro Nacional de Investigaciones Cardiovasculares (CNIC), Madrid, Spain; ^2^Unidad de Investigación Neurovascular, Departamento de Farmacología, Facultad de Medicina, Universidad Complutense de Madrid (UCM), Madrid, Spain; ^3^Instituto Universitario de Investigación en Neuroquímica (IUIN), Universidad Complutense de Madrid (UCM), Madrid, Spain; ^4^Departamento de Bioquímica y Biología Molecular, Facultad de Ciencias Químicas, Universidad Complutense de Madrid (UCM), Madrid, Spain; ^5^Instituto de Investigación Hospital 12 de Octubre (i+12), Madrid, Spain

**Keywords:** stroke, adult neurogenesis, hippocampus, SVZ, SGZ, aberrant, cognitive impairment

## Abstract

The substantial clinical burden and disability after stroke injury urges the need to explore therapeutic solutions. Recent compelling evidence supports that neurogenesis persists in the adult mammalian brain and is amenable to regulation in both physiological and pathological situations. Its ability to generate new neurons implies a potential to contribute to recovery after brain injury. However, post-stroke neurogenic response may have different functional consequences. On the one hand, the capacity of newborn neurons to replenish the damaged tissue may be limited. In addition, aberrant forms of neurogenesis have been identified in several insult settings. All these data suggest that adult neurogenesis is at a crossroads between the physiological and the pathological regulation of the neurological function in the injured central nervous system (CNS). Given the complexity of the CNS together with its interaction with the periphery, we ultimately lack in-depth understanding of the key cell types, cell–cell interactions, and molecular pathways involved in the neurogenic response after brain damage and their positive or otherwise deleterious impact. Here we will review the evidence on the stroke-induced neurogenic response and on its potential repercussions on functional outcome. First, we will briefly describe subventricular zone (SVZ) neurogenesis after stroke beside the main evidence supporting its positive role on functional restoration after stroke. Then, we will focus on hippocampal subgranular zone (SGZ) neurogenesis due to the relevance of hippocampus in cognitive functions; we will outline compelling evidence that supports that, after stroke, SGZ neurogenesis may adopt a maladaptive plasticity response further contributing to the development of post-stroke cognitive impairment and dementia. Finally, we will discuss the therapeutic potential of specific steps in the neurogenic cascade that might ameliorate brain malfunctioning and the development of post-stroke cognitive impairment in the chronic phase.

## Introduction

Stroke is a major cause of death and disability worldwide (Feigin et al., 2020). Clinical interventions to restore blood flow, mechanical or pharmacological clot removal, are the only two therapies currently approved for patient use. Both treatments are limited to the acute phase of the disease: rtPA can be given only within 4.5 h of stroke onset (Gilligan et al., 2005; Krause et al., 2019), while the window for thrombectomy has recently been extended (Campbell et al., 2019) but is still narrow. However, advances in prevention and healthcare have progressively reduced stroke mortality (Benjamin et al., 2017) and, as a consequence, stroke can now be considered a chronically disabling disease, with many stroke survivors displaying a variety of motor, cognitive, and psychiatric deficits long-term after stroke onset. These clinical manifestations depend on several factors which include extension and region of the injured brain, timing and also possible therapeutic interventions (Iadecola, 2013).

The brain has an intrinsic capability to self-repair after stroke (Zhao and Willing, 2018) and, in fact, some of these deficits, largely motor ones, show a spontaneous recovery along the chronic phase (Nakayama et al., 1994; Jørgensen et al., 1995; Kwakkel et al., 2003; Kwakkel and Kollen, 2013; Cassidy and Cramer, 2017). Indeed, in response to an acute injury, the brain displays a high degree of plasticity to reorganize its function and structure at different levels, from molecular, cellular and behavioral mechanisms, to changes in anatomy, neurochemistry and, importantly, in the generation of new neurons by neurogenesis. In this context, stroke has been reported to drive a neurogenic burst with affects the two adult neurogenic niches, the subventricular zone (SVZ) of the lateral ventricles and the subgranular zone (SGZ) of the dentate gyrus (DG) of the hippocampus (Arvidsson et al., 2002; Parent et al., 2002; Parent, 2003; Thored et al., 2006; Kernie and Parent, 2010). These exciting findings suggested that the neurogenic burst is an adaptive response for promoting brain recovery and replacing lost neurons. However, it may not be always so. Since the neurogenic cascade is a highly regulated process, exposure to the ischemic environment may result in multiple dysfunctional outcomes. In fact, it has been proposed that, maladaptive neurogenesis could in some instances contribute as a secondary process to brain malfunctioning.

In contrast with the frequent spontaneous motor recovery during the chronic phase, cognitive function tends to worsen long-term after stroke (Levine et al., 2015; Mijajlović et al., 2017). As such, one of the most troubling late complications in stroke patients is the prevalence of the so-called post-stroke cognitive impairment, which involves a series of syndromes that range from mild cognitive impairment to the most severe form, post-stroke dementia, characterized by alterations in different cognitive domains including attention, executive function, language, orientation, and memory. In fact, more than one-third of patients may develop cognitive impairment or even dementia after stroke (Tatemichi et al., 1994; Pendlebury and Rothwell, 2009; Brainin et al., 2015). However, mechanisms underlying post-stroke cognitive impairment and dementia remain quite unknown (Brainin et al., 2015; Mijajlović et al., 2017). Although cognitive deficits after stroke are considered size and location-dependent (Al-Qazzaz et al., 2014; Zhao et al., 2018), studies in both humans and animals provide evidence supporting that brain areas far from the ischemic insult and primarily undamaged may be involved; for instance, stroke may impair the hippocampal function and promote a memory decline even when the primary brain injury does not affect the temporal lobe (Prins et al., 2005; Blum et al., 2012). Still, little is known about the pathophysiological mechanisms through which ischemic infarcts may cause a secondary alteration of the function of distal areas. Several mechanisms have been proposed to account for the variety of secondary changes later after stroke, in susceptible regions such as the hippocampus, that may participate in the development of post-stroke cognitive dementia, such as a low grade chronic hypoxia, an increase in oxidative stress (Li et al., 2013; Ma et al., 2013), chronic activation of the inflammatory response, a secondary dysfunction of the vasculature altering the blood-brain barrier (BBB) or a damage into the white matter (demyelination, axonal loss and degeneration of oligodendrocytes). An interesting hypothesis is that maladaptive hippocampal SGZ neurogenesis is also a major contributor to the development of dementia after stroke (Wang et al., 2010; Candelario-Jalil et al., 2011).

## SVZ Neurogenesis in Stroke: A Potential Mechanism for Self-Repair?

In physiological situations, newborn neurons generated in the SVZ migrate through the rostral migration stream (RMS) toward the olfactory bulb (OB) where they finally differentiate into interneurons to contribute to olfactory functions (Alvarez-Buylla and Garcia-Verdugo, 2002; Ming and Song, 2011). An amount of experimental evidence supports that, in pathological situations such as an ischemic stroke, the brain activates a process of endogenous self-repair and repopulation of the damaged area by stimulating SVZ neurogenesis (Lindvall and Kokaia, 2015).

The enhancement of SVZ neurogenesis after global ischemia was first described in 1998 (Liu et al., 1998). After this initial work, many studies confirmed this finding in both rodents and humans (Eriksson et al., 1998; Jin et al., 2001; Zhang et al., 2001). Post-stroke SVZ neurogenesis has been widely studied in experimental models of cerebral ischemia with striatal affectation, such as the intraluminal middle cerebral artery occlusion (MCAO) in rodents, which shows a clear temporal profile of the different neurogenic steps (Parent et al., 2002; Parent, 2003; Kernie and Parent, 2010): in this model, the literature describes an increase in the proliferation of SVZ precursors that migrate to the lesion site and differentiate to functional neurons around the infarct (Jin et al., 2001; Zhang et al., 2001, 2004a,b; Arvidsson et al., 2002; Parent et al., 2002; Parent, 2003; Thored et al., 2006; Kernie and Parent, 2010; Lindvall and Kokaia, 2015). This increase, produced by a shortening of the cell cycle, is transient, starts 2 days after stroke onset, reaches a maximum in 1–2 weeks after the beginning of the damage (Jin et al., 2001; Zhang et al., 2001, 2004a,b; Arvidsson et al., 2002), recovering its basal levels over six weeks after the damage (Thored et al., 2006). In addition to changes in cell cycle, it has been reported that stroke transiently changes the division of neural stem cells (NSCs) from asymmetric to symmetric, thus increasing their stock in the SVZ (Zhang et al., 2004a,b).

In a longitudinal analysis performed on a mouse model of cortical ischemia by permanent MCAO induced by ligature, we found that cortical stroke has a triphasic effect on the total number of cells in proliferation at the SVZ, first with an early acute reduction of proliferation on post-stroke day 1, a second slow increase with a maximum on post-stroke day 14 and, finally, a reduction of proliferating cells at 28 days after ischemia onset. Our results also showed that this process was bilateral in all the conditions and at all the time points studied, supporting an important effect of ventricular cerebrospinal fluid (CSF) composition at this level (Palma-Tortosa et al., 2017). This bilaterality in the SVZ had also been described after cerebral ischemia by intraluminal MCAO (Jin et al., 2001). Related to this, our group has recently demonstrated that TLR4 activation with ligands such as high mobility group box 1 (HMGB1), which is released into the CSF after stroke, drives increased proliferation of neural progenitor cells (NPCs) in this setting, while also promoting differentiation of type-C progenitor cells into migrating neuroblasts (Palma-Tortosa et al., 2019).

SVZ has been identified as the main source of neuroblasts generated after stroke. Neuroblasts migrate ectopically from the pre-defined RMS to the injured area (Jin et al., 2001; Zhang et al., 2001; Arvidsson et al., 2002), a process which is described in several animal models (Christie and Turnley, 2012; Moraga et al., 2015; Bravo-Ferrer et al., 2017) and also in human studies (Minger et al., 2007; Martí-Fàbregas et al., 2010). Depending on where the damaged area is located, striatum or cortex, the ectopic migration routes differ in the path followed: whereas neuroblasts migrating into the striatum do so directly from the SVZ, adjacent to the striatum, neuroblasts traveling into the ischemic cortex do so through the corpus callosum (Ohab and Carmichael, 2008). This ectopic migration begins 3-4 days after stroke onset, is maintained until approximately 4 months of the damage (Thored et al., 2006), and is observed, similarly to the migration through RMS, as chains of neuroblasts with elongated cell bodies associated with astrocytes and blood vessels (Yamashita et al., 2006). The damaged brain tissue appears to be the main responsible in the redirection of neuroblasts. In this context, SVZ neural stem cells may receive damage-induced stimuli by two ways, either through changes in CSF composition, and/or through the diffusion of signals from the injured area to the SVZ by the damaged parenchyma or blood vessels. In this context, several molecular signals and cellular interactions that re-direct neuroblasts toward the ischemic region have been described (Young et al., 2011). For instance, inflammatory cells located in the infarct may secrete several types of chemoattractants such as stromal-derived factor 1α (SDF-1α) and monocyte chemoattractant factor 1 (MCP-1), prompting neuroblasts to migrate up chemotactic gradients along blood vessels and astrocytic processes toward the injury. Intrinsic changes of the neuroblasts in combination with an active process of tissue remodeling including the generation of new blood vessels are necessary to provide a scaffold that allows neuroblasts to reach the infarct.

Interestingly, in our longitudinal study, we also analyzed the physiological, eutopic RMS route (from the SVZ to the OB) (Palma-Tortosa et al., 2017). Our results revealed an important increase in this migration but only 24 h after the insult, with a recovery to basal levels from 2 to 28 days after stroke. This enhanced eutopic migration might explain the early reduction in the number of proliferating cells at the SVZ during the first hours after the insult. On its turn, the ectopic, damage-induced migration showed an increase from day 14 after the insult (Palma-Tortosa et al., 2017). In contrast with the effects of stroke on the proliferation in the SVZ, migration is unilateral, possibly due to cues originating from the infarcted and/or peri-infarct area, as already demonstrated (Lee et al., 2006; Bagley and Belluscio, 2010).

A great deal of evidence supports that post-stroke neurogenesis in the SVZ could be involved in the functional improvement that stroke patients undergo during the first months after the establishment of the lesion (Lindvall and Kokaia, 2015). SVZ newborn neurons after stroke could contribute to this improvement in different ways. For instance, newly generated neuroblasts may migrate toward the ischemic boundary region, where they can differentiate into fully mature neurons to replace lost neurons. However, despite the clear increase in neuroblast proliferation and migration after stroke, only a very small percentage of immature neurons reaches the damaged area and only around a 0.2% of these immature neurons fully maturate and integrate into the infarcted region (Arvidsson et al., 2002). This may be due to different factors, such as a hostile inflammatory environment due to ischemia, a deficit of functional connections and/or necessary trophic support (Ming and Song, 2011). Interestingly, an alternative/additional beneficial effect may be derived from the fact that neuroblasts have also been described to form new astrocytes that contribute to the formation of the glial scar, to protect neurons from glutamate-induced excitotoxicity or even to release neurotrophic factors that contribute to tissue repair (Arvidsson et al., 2002; Zhang et al., 2004a,b; Thored et al., 2006; Jin et al., 2010; Butti et al., 2012; Wang et al., 2012; Faiz et al., 2015). Consistent with this positive role, post-stroke SVZ neurogenesis inhibition after stroke impedes recovery after ischemia and exacerbates neurological deficits (Jin et al., 2010), whereas transplantation of neural precursor cells causes a neurological improvement indicating that this process participates in post-stroke recovery (Bacigaluppi et al., 2009). All this evidence therefore supports that post-stroke SVZ neurogenesis could be an adaptive brain plasticity process implicated in restoring brain functionality. However, for an efficient repair, the neurorestorative potential of the SVZ neurogenesis after stroke should probably be combined with the promotion of other regenerative processes that, together, will enhance the survival and integration of newborn neurons into the damaged neuronal networks.

## The Hippocampal Neurogenic Burst After Stroke: A Maladaptive Response Promoting Hippocampal Malfunctioning?

According to the literature, the SGZ in the hippocampus is the other neurogenic region of the adult brain where neurons are continuously generated throughout life. New granule cells are born in the SGZ of the DG, from where they migrate into the granule cell layer (GCL) and become functionally integrated into neuronal networks. The generation and integration of these newborn neurons seems to be strictly regulated, which is of paramount importance considering that hippocampal neurogenesis is a fundamental process for the formation and retrieval of spatial memories and, specifically, for hippocampal pattern separation (Zhao et al., 2008; Sahay et al., 2011; Akers et al., 2014; Rangel et al., 2014; Kempermann et al., 2015; Gonçalves et al., 2016).

After brain ischemia, the proliferation and differentiation of neuronal progenitors in the DG is also strongly stimulated, leading to a significant increase in neurogenesis (Liu et al., 1998; Arvidsson et al., 2001a,b; Kernie and Parent, 2010). In experimental models of global cerebral ischemia, an approximately 10-fold increase in the SGZ proliferation has been observed (Takagi et al., 1999; Kee et al., 2001; Yagita et al., 2001). This increased proliferation has also been detected after focal ischemia using the MCAO models by either permanent or transient occlusion of the middle cerebral artery (Jin et al., 2001; Türeyen et al., 2004). Therefore, even remote cortical infarcts not affecting the hippocampal formation are able to promote a significant augmentation in hippocampal neurogenesis (Keiner et al., 2010; Walter et al., 2010; Cuartero et al., 2019). Importantly, the increased neurogenic response after ischemia is observed, more or less at the same extent, in both ipsilateral and contralateral sides (Jin et al., 2001; Takasawa et al., 2002; Cuartero et al., 2019), as previously commented for the SVZ. In general, this increase in DG proliferation starts approximately one week after stroke, peaks at days 10 to 14 depending of the MCAO model, and returns to basal levels within several weeks (4-5 weeks) after onset. Although many of the new cells die, the majority of surviving cells are believed to adopt a neuronal fate in a rate similar to that observed in physiological conditions (Kempermann et al., 2015): at later times after stroke onset, they differentiate into granular cells, moving from the SGZ to the GCL and shifting their expression from immature markers like doublecortin (DCX) to mature neuronal markers, like calbindin and NeuN (Kee et al., 2001; Sharp et al., 2002; Tanaka et al., 2004), initially suggesting that hippocampal newborn neurons in the ischemic brain follow a time course of neuronal maturation similar to that described in physiological conditions.

Therefore, stroke increases the production of new neurons in the SGZ, but how the hippocampal neurogenic response influences disease outcome, positively or negatively, is still controversial. In global cerebral ischemia models, in which a massive death is observed in the hippocampus, new hippocampal CA1 neurons are detected after the injury (Nakatomi et al., 2002; Daval et al., 2004; Schmidt-Hieber et al., 2004; Bendel et al., 2005; Oya et al., 2009; Wojcik et al., 2009). Although the origin of these new CA1 neurons is not very clear, some studies have suggested that neuroblasts migrating from both the SGZ and the posterior periventricular region could be the source of these new CA1 pyramidal neurons (Nakatomi et al., 2002; Oya et al., 2009; Khodanovich et al., 2018). But the fact is that, after focal ischemia, newborn neurons in the DG do not have the capacity to migrate toward the ischemic boundary region, as observed for SVZ-derived neuroblasts. Thus, SGZ neuroblasts generated after stroke cannot replace lost neurons or promote regeneration by locally secreting trophic factors in the injured area. Instead, these newborn granule cells integrate into the granular cell layer where they will contribute to the hippocampal function. The question is whether this higher number of new granule neurons positively correlate with a better post-stroke outcome. As we will discuss in the next sections, recent evidence indicates that this may not be the case. Although the hippocampal neurogenic burst could be in principle a compensatory response, a significant proportion of newborn neurons display abnormal properties and become aberrantly integrated into the pre-existing hippocampal circuits. Therefore, adding more neurons with altered aberrant features in the DG might compromise the normal functioning of the global hippocampal network.

## Post-Stroke Neurogenesis Regulation: Internal Checkpoints and Extrinsic Factors

Neurogenesis can be controlled at different steps including NSC maintenance, proliferation, fate specification and differentiation, migration, fully maturation and, finally, newborn neuron integration into the local circuitry (Gage, 2000; Llorens-Bobadilla et al., 2015). All these stages are strictly regulated by intrinsic and extrinsic mechanisms which together determine the outcome of the neurogenic process (Faigle and Song, 2013; Bjornsson et al., 2015; Shin et al., 2015). Therefore, after a pathological insult like a cerebral ischemia, alterations in both intrinsic and extrinsic programs might contribute to the different steps of the neurogenic response in the DG.

### Intrinsic Modulators: The Heterogeneous Nature of NSCs

For hippocampal neurogenesis, the number and the type of NSCs/NPCs are key regulators in steady-state conditions. NSCs are located in the SGZ of the DG, at the interface of the hilus and the granular cell layer. Through different intermediate progenitors, they finally generate immature neuroblasts that eventually convert into young neurons (Kempermann et al., 2004, 2015; Ming and Song, 2011). Prototypical NSCs (also called type-1 or radial-glia-like progenitors) are mostly quiescent with only a few of them being mitotic. Upon activation, type-1 mainly divides asymmetrically to give rise to another NSC and a transiently amplifying population of intermediate neuronal precursors (type-2 cells). Type-2 progenitors represent an important stage of clonal expansion and linage choice: they prevalently use a symmetric division mode (Encinas et al., 2011; Pilz et al., 2018) and comprise cell states that mark the transition from a glial/stem-like phenotype (type-2a) to a neuronal phenotype (type-2b) (Steiner et al., 2006). Type-2b progenitors then generate proliferating neuroblasts (type-3 cells) that, after migrating a short distance into the granule cell layer, stop being proliferative to become immature neurons (Ming and Song, 2011).

In the context of cerebral ischemia, several studies have characterized the contribution of the different types of NSCs/NPCs to the increased post-stroke proliferation (Keiner et al., 2010; Walter et al., 2010). Six hours after the induction of cortical infarcts, an increase in the proliferation of type-1 and type-2a cells has been detected. In addition, type-2b and type-3 cells also showed increased proliferation 24–72 h after MCAO (Keiner et al., 2010; Walter et al., 2010). Following MCAO, the maximal number of proliferating cells was found after seven to fourteen days and then, proliferative activity decreased 2–5 weeks after the lesion. Therefore, it would seem that this expansion might be due, in part, to increased type-1 NSCs recruitment out of quiescence after stroke. However, this picture may not be so simple since recent studies suggest that NSCs are functionally heterogeneous and plastic in nature depending on their microenvironment. In physiological situations or even in normal aging, different kinds of hippocampal type-1 or radial-glia-like progenitors, called type α-, β-, and Ω-cells, can be distinguished by unique morphological features, specific expression markers and by their proliferative capacity and quiescence (Gebara et al., 2016; Martín-Suárez et al., 2019). Further evidence for this heterogeneity comes from recent single-cell RNA sequencing studies which also shed light on the identity of NSCs residing in the SVZ (Llorens-Bobadilla et al., 2015; Luo et al., 2015; Dulken et al., 2017; Zywitza et al., 2018; Mizrak et al., 2020) or the SGZ (Shin et al., 2015; Artegiani et al., 2017; Berg et al., 2019; Bottes et al., 2020). These studies have provided important clues which reflect the heterogeneous nature of NSCs and NPCs in both niches but also suggest distinctive features of NSCs in the two neurogenic regions. In the SVZ, several NSC and progenitor subpopulations can be defined by the expression of specific markers. However, in the SGZ niche, NSCs seem to be so heterogeneous that discrete subpopulations cannot be identified; they rather represent a heterogeneous cellular continuum that progressively downregulates genes shared with astrocytes involved in quiescence while they upregulate activation genes.

An important point is whether this NSC heterogeneity also exists after stroke, and even more, if all NSCs are equally activated or, if on the contrary, just a subpopulation of them is sensitive to the ischemic stimulus. Although this has not been explored yet in the post-stroke hippocampus, some recent data may suggest that NSC heterogeneity exists in the hippocampus after some pathological situations like epileptic disorders, or in the SVZ after ischemic injury. In the first scenario, by performing intrahippocampal injections of low and high doses of kainic acid (KA) to model epileptiform activity (EA) or mesial temporal lobe epilepsy (MTLE), Sierra et al. (2015) demonstrated that the levels of neuronal hyperexcitation provoke a differential dramatic shift in the function and morphology of hippocampal NSCs. Although these models promoted both an increase in NSC activation and, interestingly, a long-term NSC depletion, they elicited different self-renewal modes. Thus, in the MTLE model, upon hyperactivation, NSCs develop a hypertrophic and multibranched reactive phenotype, which makes them enter into the cell cycle in larger numbers, but also produces a change in the way that they divide. Indeed, they display a symmetric division and, instead of maintaining the neural progenitor pool, they contribute to increase astrogliosis by generating reactive astrocytes as main daughter cells. On the contrary, neuronal hyperactivity in the EA model induces augmented NSCs activation without developing a reactive phenotype or changing to a symmetric division and, then, the generation of newborn neurons is enhanced. In the context of cerebral ischemia, a process similar to the one described for the MTLE model has been observed for SVZ NSCs. NSCs can abandon neurogenesis almost completely and transform into reactive NSCs that migrate toward the damaged cortex and mostly generate reactive astrocytes (Faiz et al., 2015). Along this line, by using single cell RNA-seq, Llorens-Bobadilla et al. (2015) demonstrated that, after stroke, NSCs in the SVZ display different states with unique molecular signatures and that, importantly, exhibited differential responses to the ischemic insult. They found that dormant and quiescent NSCs are more responsive to an injured environment. Interestingly, they also found that, for instance, only a subset of NSCs responds to interferon-γ (IFNγ). This could imply that multiple molecularly distinct groups of NSCs coexist in the SVZ neurogenic niche, with the ability to respond differently to pathological stimuli. In addition, this heterogeneous response might also be influenced by temporal dynamics or even by regional differences within the niche (Mizrak et al., 2019). The question arising is: *are similar mechanisms guiding a heterogeneous SGZ NSCs activation upon ischemic injury?* Further studies using single cell transcriptomics are required to deepen our knowledge on the heterogeneous nature of SVZ and SGZ neural precursor population after stroke. These studies will provide insights on the intrinsic regulatory mechanisms controlling NSCs activation upon stroke in both niches and about the type of stimuli that mediates NSCs priming and activation.

### Extrinsic Modulators

Apart from the features of neurogenesis which are intrinsic to the cells that populate the niche, the microenvironment plays a fundamental role in guiding the neurogenic process. Quiescent NSCs in both SGZ and SVZ display an enriched expression of genes related to cell-cell adhesion and cell-microenvironment interaction, suggesting that intrinsic and extrinsic signals are actively involved in maintaining stem cell quiescence (Shin et al., 2015; Morizur et al., 2018). Supporting this, when transplanted into a non-neurogenic area, hippocampal neural stem cells lose their ability to differentiate into neurons (Shihabuddin et al., 2000), indicating that the intrinsic neurogenic potential of NSCs is controlled by the neurogenic niche. Therefore, local signals from the niche as well as remote signals coming, for instance, from CSF or the blood might contribute to the regulation of post-stroke neurogenesis. Subsequently, several mechanisms could account for post-stroke aberrant neurogenesis:

(1) *Mediators of post ischemic excitotoxicity or cortical spreading depression*, such as K^+^ and glutamate elevations, could be involved, as both have been implicated in an enhanced proliferation of immature cells while at the same time directing toxicity in mature cell types (Shi et al., 2007). Granule cell development is regulated by activity-dependent mechanisms, especially NMDA receptor-mediated input. Therefore, an excessive NMDA receptor activation might contribute to the increased excitatory postsynaptic currents (EPSCs) observed in newborn neurons after stroke due to postsynaptic receptor insertion (Ceanga et al., 2019).
(2) *Injury-induced circulating factors* might directly affect neural progenitors that are in physical contact with the vasculature (Mignone et al., 2004; Tavazoie et al., 2008). For instance, stroke upregulates the levels of circulating vascular endothelial growth factor (VEGF) that easily permeates the characteristic BBB of neurogenic niches. This induces local endothelial cells to increase Notch signaling that, in turn, stimulates neurogenesis (Lin et al., 2019). Another factors identified after stroke and which might directly affect NSC proliferation are, among others, fibroblast growth factor-2 (FGF-2) (Yoshimura et al., 2001, 2003), insulin-like growth factor-1 (IGF-1) (Yan et al., 2006) and brain-derived neurotrophic factor (BDNF) (Chen et al., 2005). Furthermore, several mediators like SDF-1α, MCP-1, and matrix metalloproteinases (MMP) have been implicated in influencing neuroblast positioning and extracellular matrix remodeling (Rahman et al., 2020).
(3) Another influence to be considered is the *inflammatory response* elicited by the injured brain, whereby astrocytes and microglia activation could lead to the secretion of a variety of growth factors and immune modulators able to affect progenitor proliferation and survival. In addition, several studies associate pro-inflammatory cytokines released after stroke with proliferation, migration, differentiation, and survival of neural precursors (Tobin et al., 2014). In this line, Meng et al. demonstrated that IL-6 promotes the proliferation and differentiation of NPCs in the adult SVZ, inducing functional improvement after stroke. Furthermore, the blockade of IL-1 receptor after experimental ischemic stroke resulted in functional improvement of the mice as well as in increased NSCs proliferation of NSCs. In turn, it enhanced neuroblast migration and increased the number of new neurons formed in the peri-infarct cortex (Pradillo et al., 2017).

Despite the extensive characterization of post-stroke neurogenesis in both SVZ and SGZ and of the factors involved, the exact upstream and downstream signaling pathways driving this transient increase in the neurogenic response is still unknown. In this sense, further studies are necessary to dissect each step of the neurogenic cascade after stroke, trying to answer remaining questions like: *do the same mediators act in both adult niches after stroke?; do all hippocampal NSCs equally respond to the injured brain?; does a differential microenvironment account for the morphological differences in ipsi- and contralesional newborn neurons?*

## The Aberrant Phenotype of Post-Stroke Hippocampal Newborn Neurons

### Morphological Alterations of Newborn Neurons After Stroke

In the hippocampus, the maturation stage of newborn neurons can be identified based on the expression of specific molecular markers but also by the progressive development of unique morphological features (van Praag et al., 2002; Zhao et al., 2006). Mature granule neurons generally have only one primary apical dendrite emerging from the soma which is vertically oriented toward the molecular layer (ML). Although basal dendrites can be observed in humans and non-human primates (Llorens-Martín et al., 2015), rodent mature granule cells lack basal dendrites under physiological conditions (Shapiro et al., 2005, 2007). The apical dendrite remains poorly bifurcated until it reaches the ML, where it branches extensively in order to receive inputs from the entorhinal cortex (EC) through “the perforant pathway” and establishes excitatory synapses (van Praag et al., 2002; Zhao et al., 2006; Kelsch et al., 2008). Therefore, the growth of the apical dendrite seems to be a critical factor for the correct integration of newborn neurons (Shapiro et al., 2007; Llorens-Martín et al., 2015). During maturation, newborn neurons also progressively lengthen their axons and send them toward CA3 through the mossy fiber terminals and also to the CA2 area (Zhao et al., 2006; Kohara et al., 2014). This characteristic morphology of granule neurons has been so-called Y-shape to distinguish from other morphologies found after pathological situations (Llorens-Martín et al., 2015). Indeed, the elongation, orientation, location and branching of newborn neurons in the DG are sculpted by many factors and are very vulnerable to injury. For example, in rodents, environmental enrichment and wheel-running modify the morphology and connectivity of newborn neurons (Kempermann et al., 1997; Gonçalves et al., 2016).

An “aberrant morphology” of newborn granule neurons has been described after different pathological situations (Llorens-Martín et al., 2015; Bielefeld et al., 2019). Since information coming from the EC reaches the hippocampus via the DG, abnormalities in the granule cells might have important consequences for the global hippocampal network function. The concept of aberrant neurogenesis was first described in the context of seizure-associated plasticity that leads to long-lasting structural changes in hippocampal morphology. In the setting of cerebral ischemia, we and others have characterized morphological features of newborn hippocampal neurons after different times after stroke onset. By using retroviral labeling to visualize new granule cells, it has been found that different stroke models lead to alterations of newborn neuron morphology (Niv et al., 2012; Woitke et al., 2017; Cuartero et al., 2019; Sheu et al., 2019), suggesting that integration of these abnormal neurons could promote aberrant hippocampal circuitry rearrangements and, therefore, might contribute to hippocampal cognitive deficits observed after cortical ischemia. From all newborn neurons generated after stroke, a subset displayed morphological alterations. In the studies by Niv et al. (2012) and Woitke et al. (2017), wherein retroviral infection was carried out 4 days after ischemia and the morphological evaluation approximately 6 weeks post-infection, the aberrant features of newborn neurons (5–10%) included the presence of ectopic newborn neurons, abnormal basal dendrites directed toward the hilus and an increase in dendritic complexity with a subsequent increase in the total dendritic length. Importantly, all these alterations seemed to be dependent on the initial lesion size, because the percentages of aberrant neurons in the filament MCAO model was higher than in the cortical photothrombotic model (Niv et al., 2012). We have also characterized the presence of aberrant newborn neurons after stroke by using the ligature permanent MCAO model and a different timing for retroviral infection and morphological evaluation (Cuartero et al., 2019). Thus, after GFP retrovirus infusion into the ipsilesional hippocampus 14 days after surgery and morphology evaluation 35 days post-infection, approximately 28% of the newborn neurons displayed shortening of the apical dendrite, decrease in the dendritic length, and a differential pattern of arborization. Furthermore, aberrant neurons displayed an increased degree of branching in the proximal domain of the dendritic tree and a retraction of the distal domain in the ML, which can also be observed in immature DCX^+^ neurons. Similar results, with reduction in the total dendritic length and total dendritic branches, were observed in the study of Sheu and collaborators (Sheu et al., 2019). In this work, retroviral infection was performed 7 days after the filament MCAO model, and mice were euthanized at different time points after retroviral labeling to assess the maturation process of the new generated neurons.

**FIGURE 1:**
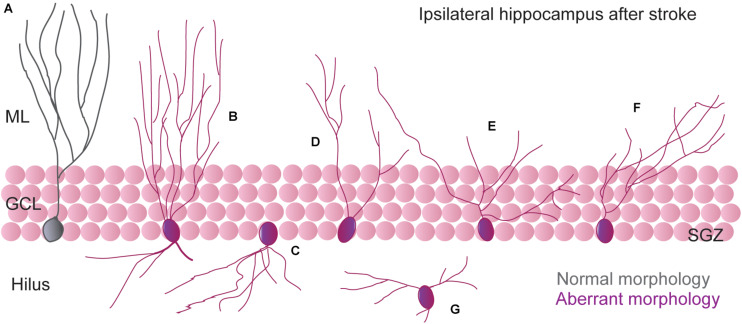
Schematic illustration of most representative aberrant dendritic morphology of newborn neurons generated in the DG after stroke. **(A)** Morphology of regular (“non-aberrant”) neurons. **(B)** Neurons with additional basal dendrites toward the hilus (bipolar cells) and an increase in dendritic complexity (Niv et al., 2012; Woitke et al., 2017). **(C)** Neurons with abnormal basal dendrites directed toward the hilus (Niv et al., 2012; Woitke et al., 2017). **(D–F)** Neurons with a dramatic reduction in apical dendrite length and an increased proximal dendritic branch density (Cuartero et al., 2019; Sheu et al., 2019). **(G)** Ectopic neurons (Niv et al., 2012; Woitke et al., 2017).

All these studies corroborate the presence of an altered morphology in part of the new granule neurons after stroke and, importantly, based on the normal presence of mushroom spines, all these studies suggest that these neurons might be stably integrated into the hippocampal network. However, the aberrant phenotype observed seems to clearly differ between different studies (Figure 1). A plausible explanation for these different phenotypes is that the generation of new aberrant neurons after stroke is a dynamic process so that, depending on the timing of infection and/or visualization, newborn neurons might display specific aberrant features. Indeed, we also tested this possibility by delivering the GFP-expressing retrovirus 35 days after stroke, when post-stroke neurogenesis had almost returned to physiological levels (Cuartero et al., 2019). In this setting, one month after infection, part of the aberrant features previously detected at earlier times points was no longer observed (such as the pattern of arborization or the reduction in mean apical dendrite length). Nevertheless, a population of newborn neurons still displayed apical dendrite growth alterations. This might suggest that aberrant neurogenesis after stroke persists at a lower intensity at later times points and, also, that the factors that induce morphological remodeling, albeit likely decreasing over time, are still present at later time points.

As commented above, the neurogenic burst after stroke affects both the ipsi- and contralesional hemispheres equally, producing an increased proliferation and generation of newborn neurons with similar temporal dynamics, suggesting that the same mediators act in both hemispheres for increasing hippocampal neurogenesis in this scenario. Therefore, if in terms of proliferation, both ipsilateral and contralateral hemispheres behave similarly, *do newborn neurons display an aberrant phenotype in the contralesional side?* We assessed this question in our study by a comparison of both ipsi- and contralesional hippocampi after stroke. Importantly, we found that a different remodeling process seems to occur simultaneously at each side of the hippocampus after ischemic stroke. Indeed, by analyzing immature and mature newborn neurons, we found a different phenotype of newborn neurons at the ipsilesional when compared with the contralesional hippocampus that seems to affect both synaptic inputs and outputs. First, contralesional neurons display an elongation of apical dendrite and, consequently, an increase in the distal dendrite branch that could have important consequences for incoming information from the EC. Second, our data suggest that stroke also promotes a differential bilateral remodeling in the connectivity between DG and CA3. Each new cell in the DG projects a mossy fiber that reaches the CA3 region within approximately 2 weeks, contacting with 11–15 pyramidal cells (Toni et al., 2008). Interestingly, in CA3, we observed a hyper- or a hypo-integration pattern of immature newborn neurons, at the contra- and ipsilesional hemispheres, respectively. These data could imply an increased synaptic rearrangement in the contralesional CA3, which could coexist or replace the previous DG-CA3 synapses. Although we did not confirm this result in mature neurons, newly generated neurons transiently display enhanced synaptic plasticity (Schmidt-Hieber et al., 2004; Ge et al., 2007b; Dieni et al., 2016), suggesting that immature new neurons may also have the ability to deprive pre-existing synapses, which may have therefore important consequences on the hippocampal function.

### Altered Electrophysiological Properties of New Granule Cells After Stroke

Although the total number of newborn neurons in the adult hippocampus is very small compared to that of the granule cells being born during development, new immature granular cells display specific cellular properties that differentiate them from the mature ones (Schmidt-Hieber et al., 2004; Ge et al., 2007b; Ming and Song, 2011; Dieni et al., 2016; Gonçalves et al., 2016). For example, immature newborn neurons exhibit hyperexcitability in the first month after birth (Mongiat et al., 2009; Dieni et al., 2013, 2016). As a consequence, they are very efficient in generating action potentials (Marín-Burgin et al., 2012). Furthermore, immature granular neurons also display enhanced synaptic plasticity with a lower threshold for the induction of long-term potentiation (LTP) upon perforant pathway stimulation (Schmidt-Hieber et al., 2004; Ge et al., 2007b) and enhanced LTP at mossy fiber connections into CA3 pyramidal neurons (Gu et al., 2012). This enhanced synaptic plasticity is partially due to a reduction in the GABAergic inhibition in immature neurons (Ge et al., 2007a).

Therefore, just by increasing the number of newborn neurons, as occurs after ischemia, important consequences for the hippocampal network might be expected. However, the presence of newborn neurons with an altered morphology might be an indication that stroke not only promotes an augmentation in the newborn neurons but also alters some of their main unique features. Then, *does stroke also alter intrinsic electrophysiological properties of hippocampal newborn neurons?* Recently, Ceanga et al. (2019) aimed to answer this question by evaluating how stroke influences the electrophysiological development of newborn hippocampal neurons. Using doublecortin-dsRed mice to label immature neurons, they found that stroke altered the intrinsic electrophysiological properties of a subset of newborn neurons, with an increase in the number of hyperexcitable immature neurons as earlier as 2 weeks after stroke onset. Of note, these young neurons displayed features that resembled those observed in the mature ones, therefore suggesting that stroke causes an accelerated maturation of new granule cells to become incorporated into the hippocampal network. Under physiological conditions, intrinsic maturation and synaptic excitability of newborn neurons are tightly coordinated processes. In fact, immature granule neurons are hyperexcitable in part to compensate for the low excitatory innervation (Ming and Song, 2011; Toni and Schinder, 2015). However, after stroke, immature neurons displayed an increase in the amplitude of spontaneous and miniature EPSCs indicating that immature newborn neurons also receive increased excitatory inputs. Therefore, stroke seems to result in an uncoupling of synaptic hyperexcitability which further potentiates the intrinsic hyperexcitability of immature newborn neurons. Of note, a fundamental function of immature newborn neurons is the inhibition of mature granule cells by regulating GABAergic interneurons that modulate mature granule cells activity (Drew et al., 2016). Consequently, increasing hippocampal newborn neurons after cerebral ischemia could be a mechanism for reducing hyperactivity by driving a local inhibition of mature granule cells. However, newborn granule cells are still hyperexcitable despite receiving large excitatory inputs (Ming and Song, 2011). Hence, stroke results in an accelerated development of intrinsically hyperexcitable new granule cells that receive unusually large excitatory inputs, therefore promoting higher hyperactivation of the hippocampal circuits. Interestingly, the morphological features of the immature neurons which displayed altered intrinsic patters of maturation after stroke were completely normal. This fact indicates that, after cerebral ischemia, normal neuronal morphology does not preclude disturbances of intrinsic excitability. Therefore, both normal and aberrant morphological newborn neurons could promote hippocampal malfunctioning after stroke.

## More New Granule Cells After Stroke: A Maladaptive Response?

The hippocampus is one of the main adult brain regions implicated in cognitive functions. Through adult neurogenesis, newborn neurons are continually added to the hippocampal circuits, contributing to the encoding of new hippocampus-dependent memories (Jørgensen et al., 1995). However, neuronal integration also produces remodeling of pre-existing hippocampal network and, therefore, increasing neurogenesis may also promote the destabilization and even the clearance of previous stored memories (Nakayama et al., 1994). Numerous examples have been provided for the role of physiological DG neurogenesis in hippocampus-dependent learning and memory processes (Kempermann et al., 2015; Gonçalves et al., 2016). Under physiological conditions, higher rates of neurogenesis in the dentate gyrus may improve cognition (Sahay et al., 2011) and may also mediate forgetting of previously acquired memories (Akers et al., 2014), but the same may not necessarily be true under a pathological situation.

Although aberrant neurogenesis has been proposed as contributing to cognitive impairment in different pathological situations, such as epilepsy, schizophrenia, and neurodegenerative diseases (Zhao et al., 2008; Ming and Song, 2011; Aimone et al., 2014; Schreglmann et al., 2015), in the context of cerebral ischemia, the functional consequences of hippocampal neurogenesis and its relation with post-stroke cognitive impairment is still controversial. Part of this controversy is probably due to differences in the cerebral ischemia model used, in the temporal experimental design, as well as in the behavioral paradigm chosen for the evaluation of cognitive function. The positive role of hippocampal neurogenesis in stroke came mostly from studies in which the ischemic injury, caused by both global (Nakatomi et al., 2002; Bendel et al., 2005) or focal ischemia models (Luo et al., 2007; Li et al., 2009; Kernie and Parent, 2010), directly affects the hippocampus, although some positive effects have also been described in MCAO models without hippocampal alteration. In these settings, maneuvers that increase hippocampal neurogenesis such as different types of rehabilitative training (for instance, running), have also shown to correlate with better functional outcomes (Wurm et al., 2007; van Praag, 2008; Zhao et al., 2008). On the contrary, as commented above, we and others have demonstrated that some newborn neurons after stroke display aberrant functional and morphological features (Niv et al., 2012; Woitke et al., 2017; Ceanga et al., 2019; Cuartero et al., 2019; Sheu et al., 2019), and also that the occurrence of aberrant neurogenesis correlates with hippocampus-dependent deficits after focal cortical stroke. Supporting this, interventions directed to increase neurogenesis after stroke, like free running, exacerbate cognitive impairments while increasing hippocampal neurogenesis. Although it is true that hippocampal aberrant neurogenesis seems to be transient and decreases over time (Cuartero et al., 2019), the important fraction of aberrant neurons generated during the stroke-induced neurogenic burst will likely remain abnormally integrated into the hippocampal circuits. Therefore, aberrant neurogenesis would have long lasting effects after stroke.

In order to clarify the role of hippocampal neurogenesis after stroke, different strategies have been used to suppress post-stroke neurogenesis. Several studies have suggested that a decrease in neurogenesis has a negative impact on cognitive function after cerebral ischemia, either using brain ionized radiation (Raber et al., 2004; Zhu et al., 2009; Shiromoto et al., 2017) or anti-mitotic agents like cytosine-β-D-arabinofuranoside to eliminate NPCs (Arvidsson et al., 2002; Zhang et al., 2004a). However, both depletion strategies might promote a non-specific elimination of all dividing cells, affecting different cells types like microglia, astrocytes or endothelial cells in addition to neural progenitor cells. In order to target NSCs/NPCs more specifically, different genetic models have been used in the context of ischemia. In the studies by Li and collaborators (Jin et al., 2010; Wang et al., 2012), authors generated a transgenic mouse expressing herpes simplex virus-1 thymidine kinase (HSV-TK) under the control of the DCX promoter, in which ganciclovir treatment produces the depletion of DCX-expressing cells in the SVZ and the SGZ. Supporting a neuroprotective role of neurogenesis after stroke, they found that depletion of DCX cells increases infarct volume, promotes an exacerbation of short-term sensory motor deficits and impairs long-term recovery after stroke, although they did not evaluate post-stroke cognitive function. The causal relationship between neurogenesis and the post-stroke cognitive function was evaluated by Sun and cols. (Sun et al., 2013) by using a transgenic mouse in which HSV-TK was under the control of the nestin promoter. In this case, neurogenesis inhibition impaired spatial learning and memory in the Barnes maze after stroke. Although all together these studies suggest that post-stroke neurogenesis is necessary for recovery of cognitive function after stroke, several considerations must be taken in to account. First, the genetic models used in these studies targeted both post-stroke SVZ and SGZ neurogenesis; therefore, confounding results might come from a differential contribution of SVZ and SGZ neurogenesis in recovery after stroke, for instance, by affecting the infarct volume size, since progenitors from the SVZ are known to be recruited to the ischemic lesion as commented above. Most importantly, in both studies, neurogenesis suppression was performed prior to the MCAO procedure, which might promote basal improper cognitive function by reducing basal neurogenesis levels when the brain is still healthy instead of blocking just post-stroke neurogenesis.

**FIGURE 2:**
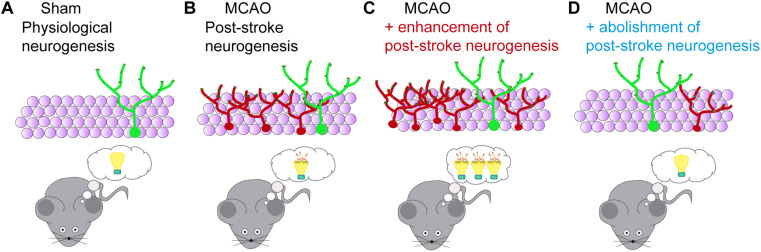
Functional consequences of modulation of post-stroke neurogenesis after stroke. **(A)** The hippocampus is one of the main adult brain regions implicated in cognitive functions. Through adult neurogenesis, newborn neurons are continually added to the hippocampal circuits, contributing to the encoding of new hippocampus-dependent memories. **(B)** Stroke-induced neurogenesis in the SGZ positively correlates with memory impairment after cerebral ischemia. **(C)** Enhancement of post-stroke neurogenesis, for instance, by running, exacerbates hippocampal cognitive deficits after ischemia. **(D)** Post-stroke memory impairment is reduced by abolishment of ischemia-induced aberrant neurogenesis (Cuartero et al., 2019).

In an attempt to solve some of these limitations, we designed a specific strategy for abolishing neurogenesis after MCAO (Cuartero et al., 2019) without altering basal neurogenesis. For such a purpose, we used nestin-Cre^*ERT2*^/NSE-DTA mice. In these mice, tamoxifen-inducible Cre is expressed by NSCs under the nestin promoter (Line 4, with display a higher recombination in the SGZ) and the loxP-STOP-loxP-IRES-DTA gene cassette is knocked into the NSE (Eno2; enolase 2) gene (Imayoshi et al., 2006, 2008). After tamoxifen treatment, Cre recombinase deletes the STOP sequence in the NSC pool. Throughout maturation, the NSE promoter becomes active, inducing the expression of the diphtheria toxin A (DTA), resulting in cellular programmed death. Thus, the generation of fully mature newborn granule neurons is dramatically decreased in these mice. Using tamoxifen administration after stroke (starting at day 7), we were able to reduce post-stroke hippocampal neurogenesis, and both proliferating cells and immature newborn neurons returned to similar levels that in sham mice. By abolishing neurogenesis, we clearly detected that MCAO mice displayed an improvement in contextual and spatial memory recall performance when tested long-term after stroke (Figure 2), supporting that the inhibition of aberrant hippocampal post-stroke neurogenesis avoids the onset of remote memory deficits after stroke (Cuartero et al., 2019), and strongly supporting the involvement of aberrant hippocampal neurogenesis in post-stroke cognitive impairment and dementia.

## Future Challenges: Targeting Post-Stroke Neurogenesis

The post-stroke neurogenic burst in both SVZ and SGZ niches may have different functional consequences in stroke outcome. While increasing SVZ neurogenesis could promote functional recovery, augmentation of hippocampal neurogenesis could further exacerbate the development of post-stroke cognitive impairment. Therefore, in order to consider neurogenesis as a potential therapeutic post-stroke target, it would be necessary to design specific strategies in a *niche-dependent fashion*. Thus, instead of a complete modulation of the post-stroke neurogenic response, strategies should be directed to enhance or inhibit specific steps of the post-stroke neurogenesis.

One potential specific maneuver after stroke might be the enhancement of the survival and integration of SVZ-derived neuroblasts in the injured brain. In this context, given the very low degree of survival of neuroblasts that migrate to the damaged tissue and their low differentiation into mature neurons, the major emphasis from a therapeutic standpoint should probably be put on approaches that improve these processes. Thus, different strategies have focused on trying to regenerate damaged tissue by either increasing endogenous neurogenesis (Wu et al., 2017) or by stem cell therapy or transplantation, resorting to a variety of stem cell sources (Bang et al., 2016; Gervois et al., 2016; Hess et al., 2017).

On its hand, at the level of the SGZ, strategies should focus toward the prevention of the cases of maladaptive neurogenesis leading to aberrant morphologies of newborn granule neurons and their detrimental consequences.

Although we are still a long way from having a whole picture of the post-stroke neurogenesis process, current investigations will likely allow us to dissect the multi-step neurogenic response in both niches, gaining insight on how all these processes are initiated and maintained, and paving the way to develop new therapeutic avenues for stroke patients.

## Author Contributions

MC, AG-C, and MM conceptualized the study. IL and MM were responsible for the funding acquisition. MC, AG-C, and MM contributed to the writing (original draft). MC, AG-C, CT-L, VM, EF, SV-R, JG-S, IL, and MM contributed to the writing (review and editing). All authors read and approved the final manuscript.

## Conflict of Interest

The authors declare that the research was conducted in the absence of any commercial or financial relationships that could be construed as a potential conflict of interest.
